# Post‐translocation dynamics of black‐tailed prairie dogs (*Cynomys ludovicianus*): A successful conservation and human–wildlife conflict mitigation tool

**DOI:** 10.1002/ece3.9738

**Published:** 2023-01-09

**Authors:** Noelle C. Guernsey, Patrick E. Lendrum, Lindsey Sterling Krank, Shaun M. Grassel

**Affiliations:** ^1^ Humane Society of the United States – Prairie Dog Conflict Resolution Washington DC USA; ^2^ World Wildlife Fund – Northern Great Plains Program Bozeman Montana USA; ^3^ Lower Brule Sioux Tribe Department of Wildlife, Fish, and Recreation Lower Brule South Dakota USA; ^4^ First Nations Development Institute Longmont Colorado USA

**Keywords:** black‐footed ferret, black‐tailed prairie dog, coterie, *Cynomys ludovicianus*, social behavior, translocation

## Abstract

Prairie dogs have declined by 98% throughout their range in the grasslands of North America. Translocations have been used as a conservation tool to reestablish colonies of this keystone species and to mitigate human–wildlife conflict. Understanding the behavioral responses of prairie dogs to translocation is of utmost importance to enhance the persistence of the species and for species that depend on them, including the critically endangered black‐footed ferret. In 2017 and 2018, we translocated 658 black‐tailed prairie dogs on the Lower Brule Indian Reservation in central South Dakota, USA, a black‐footed ferret recovery site. Here, we describe and evaluate the effectiveness of translocating prairie dogs into augered burrows and soft‐released within presumed coteries to reestablish colonies in previously occupied habitat. We released prairie dogs implanted with passive integrated transponders (PIT tags) and conducted recapture events approximately 1‐month and 1‐year post‐release. We hypothesized that these methods would result in a successful translocation and that prairie dogs released as coteries would remain close to where they were released because of their highly social structure. In support of these methods leading to a successful translocation, 69% of marked individuals was captured 1‐month post‐release, and 39% was captured 1‐year post‐release. Furthermore, considerable recruitment was observed with 495 unmarked juveniles captured during the 1‐year post‐release trapping event, and the reestablished colony had more than doubled in the area by 2021. Contrary to our hypothesis, yet to our knowledge a novel finding, there was greater initial movement within the colony 1‐month post‐release than expected based on recapture locations compared with the published average territory size; however, 1 year after release, most recaptured individuals were captured within the expected territory size when compared to capture locations 1‐month post‐release. This research demonstrates that while translocating prairie dogs may be socially disruptive initially, it is an important conservation tool.

## Graphical abstract

Prairie dogs have declined by 98% throughout their range in the grasslands of North America. We successfully translocated black‐tailed prairie dogs into augered burrows and soft released within presumed coteries to reestablish colonies in previously occupied habitat. Prior to establishing new territories, prairie dogs made greater initial movements then expected demonstrating that while translocating prairie dogs may be socially disruptive initially, it is an important conservation tool.

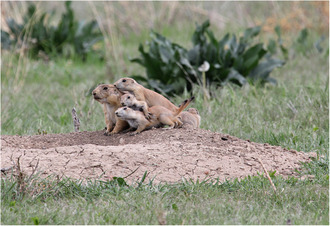

## 1 INTRODUCTION

Prairie dogs (*Cynomys* spp.) are colonial ground squirrels that have an important role as a keystone species across the North American Great Plains though their distribution has been reduced by 98% of the historical range (Hoogland, 2006a). Through their herbivory and burrowing behaviors, prairie dogs create unique habitat within the western grasslands. Clipping and grazing results in short vegetation patches often consisting of forbs and grasses, combined with the creation and maintenance of burrow networks and mounds offer habitat heterogeneity relative to grasslands uninhabited by these ecosystem engineers (Davidson et al., 2012; Whicker & Detling, 1988). Numerous animal species are associated with prairie dog colonies, including those that use the habitat created, such as burrowing owls (*Athene cunicularia*) and mountain plovers (*Chadrius montanus*); and those that prey heavily on prairie dogs, such as ferruginous hawks (*Buteo regalis*), American badgers (*Taxidea taxus*), coyotes (*Canis latrans*), and black‐footed ferrets (*Mustela nigripes*) (Clark et al., 1982; Kotliar et al., 1999). Several species that are prairie dog associates are of conservation concern, most notably of which is the endangered black‐footed ferret—an obligate predator of prairie dogs and requires extensive and relatively intact prairie dog colonies (Antolin et al., 2002).

There are five species of prairie dogs in North America, two of which are considered threatened (Utah prairie dog (*C*. *parvidens*)) or endangered (Mexican prairie dog (*C. mexicanus*)) and the remaining three species of prairie dogs (black‐tailed prairie dog (*C. ludovicianus*), white‐tailed prairie dog (*C. leucurus*), and Gunnison's prairie dog (*C. gunnisoni*)) are rare compared with historical populations (Ceballos et al., 1993; USFWS, 2000, 2012, 2013, 2017). The black‐tailed prairie dog is listed as threatened in Canada under the Species at Risk (Parks Canada Agency, 2021). Threats to prairie dogs include rangeland conversion, inadequate regulatory mechanisms resulting in direct persecution, and disease (Luce et al., 2006). Prairie dogs are highly susceptible to sylvatic plague, which is caused by a non‐native bacterium (*Yersinia pestis*) and is likely the most significant threat to the persistence of prairie dogs and many of their associated species (Antolin et al., 2002; Cully et al., 2006; Eads & Biggins, 2015; USFWS, 2000). Plague epizootics can lead to extensive landscape level impacts because colonies often can experience >90% mortality and recolonization can be slow (Cully et al., 2010; Eads & Biggins, 2015) or never reach pre‐epizootic levels (Antolin et al., 2002; Biggins & Eads, 2019, R. Matchett, personal communication).

Translocation is the human‐mediated movement of living organisms from one area, with release in another (IUCN/SSC, 2013). Developing successful translocation programs for ecologically important species experiencing defaunation has many potential benefits for conservation (Watson & Watson, 2015). Translocation offers a restoration method that can repopulate suitable habitat much more quickly than would occur naturally (Dullum et al., 2005). The reestablishment of self‐sustaining wildlife populations is challenging and improvement of approaches, including the inclusion of the social behavior of the species, have been added to the field of conservation translocation science to guide on‐the‐ground implementation (Goldenberg et al., 2019). Active restoration of prairie dog colonies through translocation can serve to contribute to conservation goals for both prairie dogs and their associated species (Dullum et al., 2005), while simultaneously offering a wildlife–human conflict mitigation opportunity (Nelson & Theimer, 2012).

Prairie dog translocations have been used as a tool for many years, with different methods that include releasing into artificial burrow systems made from corrugated, plastic drainpipe and artificial nest boxes (Bly‐Honness et al., 2004; Curtis et al., 2014; Roe & Roe, 2004), intact natural burrows (Truett et al., 2001), and burrows created with gas‐powered augers (Dullum et al., 2005). The biotic and abiotic factors of previously occupied habitat or suitable novel habitat have been important for release site selection; furthermore, site preparations that consider the behavioral ecology of prairie dogs such as group compositions, mowing vegetation to suitable height and predator deterrents have been principal considerations for recipient sites (Long et al., 2006). During translocations, prairie dogs have been hard‐released and soft‐released, which can include the use of acclimation cages and supplemental feeding (Long et al., 2006; Truett et al., 2001). Some researchers have stressed the importance of releasing prairie dogs with other members of their coteries of the source colonies due to their highly social population structure (Bly‐Honness et al., 2004; Shier, 2006). Coteries are polygynous units that typically contain one breeding male, related females, and their young that maintain a spatially discrete territory (Hoogland, 2006b). Across methods, there has been a wide range of success documented ranging from low survival rates and the failure to establish colonies to the establishment of colonies that have expanded in size and persisted (Curtis et al., 2014; Dullum et al., 2005; Roe & Roe, 2004; Shier & Owings, 2007). Choosing the ideal suite of translocation methods needs to be based on a variety of factors including but not limited to release site condition (e.g., soil type, pre‐existing burrows, etc.), prevalence of predators, history of previous extirpation and sylvatic plague mitigation tools, cultural or ecological significance of the site, and resource availability.

We evaluated the effectiveness of the translocation of black‐tailed prairie dogs to reestablish colonies in previously occupied habitat on the Lower Brule Indian Reservation in central South Dakota, USA, where prairie dogs had been absent following sylvatic plague epizootic events in 2011 and 2013. Prior to plague epizootics, the site supported a population of federally endangered black‐footed ferrets. Because of adjacent topographical features and the lack of nearby colonies of prairie dogs, natural recolonization of the site had been especially slow with annual site visits confirming the areas remained unoccupied since plague epizootic events occurred (Grassel, personal communication). To meet wildlife conservation objectives for the site, a past translocation was conducted in 2015 when 520 prairie dogs were captured elsewhere and released. The release sites were prepared by mowing vegetation and opening burrows that had partially collapsed with gas‐powered augers. Release burrows were treated with deltamethrin to control fleas and prevent mortalities from plague (Seery et al., 2003). Prairie dogs were hard‐released without the use of acclimation cages or supplemental food, coteries were not maintained, and prairie dogs failed to establish from this effort (Grassel, personal communication). While this effort implemented some of the guidelines for conservation translocations, there were aspects in the release strategy that offered the opportunity for improvements (IUCN/SSC, 2013). In the next iteration of the translocation effort, further modifications were made to incorporate potential behavioral considerations including social structure and predator avoidance.

Our primary objective was to reestablish prairie dog colonies using soft‐release methods while maintaining coterie structure and evaluate restoration success. The site had a history of being occupied and the translocation methods we employed had been successful elsewhere, therefore, we anticipated that translocated prairie dogs would successfully recolonize the site. We considered animal retention one‐month post‐release as our short‐term measure of translocation success, with success further defined as maintaining a sex ratio of adults that would allow for pup recruitment the following spring and greater than 50% of animals released recaptured. Assuming short‐term success was realized, we quantified mid‐term success 1‐year post‐release as continued animal retention, juvenile recruitment, and colony expansion, which would all signify a successful translocation. Through continued monitoring and mapping of the site, we evaluated continued success defined as site occupancy and expansion three or more years post‐release.

A secondary objective was to enhance understanding of coterie dynamics after release. As previously noted, coterie dynamics are highly complex and vary depending on the family group, sex (male or female), and age (juvenile or adult) composition, all of which influence the group size of the coterie. In turn, these complex interactions may influence the cohesion of the coterie and resiliency to disturbances. Because of the strong social structure of colonial species, we hypothesized prairie dogs would remain in the immediate area where they were released as a coterie. To complement this spatial component, we also examined whether the number of individuals in a coterie and age and sex composition influenced site retention. A better understanding of these objectives is necessary for conservation practitioners working to maintain the ecological function of North America's most imperiled biome (Comer et al., 2018), and ultimately the conservation of the prairie dog ecosystem, including contributions to the recovery of black‐footed ferrets.

## 2 METHODS

### 2.1 Study site

**FIGURE 1:**
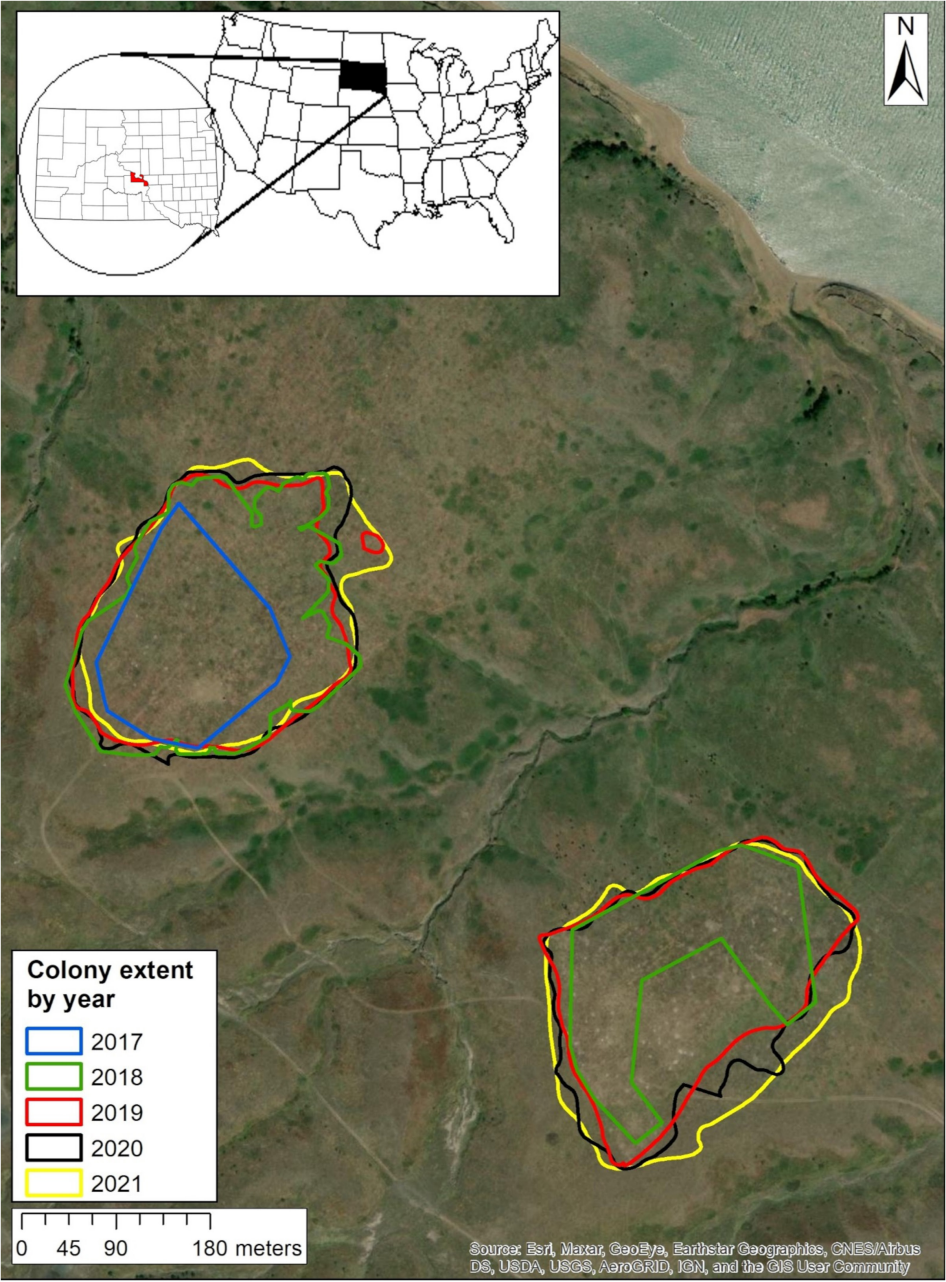
Translocation release site at the lower Brule Indian Reservation in Central South Dakota. The northern colony (Charlie's) was established in 2017, and the southern colony (Charlie's South) was established in 2018. Colony extent was mapped each year with the latest mapping in 2021.

We translocated prairie dogs to two inactive prairie dog colonies <500 m apart (Charlie's and Charlie's South) that had previously experienced plague events within the Fort Hale Bottom prairie dog complex at the Lower Brule Sioux Indian Reservation in central South Dakota, USA (43°59′N, 99°29′W; Figure 1). The site is characterized as mixed‐grass prairie with western wheatgrass (*Agropyron smithii*), green needlegrass (*Stipa viridula*), buffalograss (*Bouteloua dactyloides*), and needle‐and‐thread (*Hesperotipa comata*). Soils at depths up to a meter were dominated by silty loam (USDA‐NRCS, Ecological Site Description R063AY010SD, n.d.). The sites selected to receive translocated prairie dogs were areas that could be distinguished from the surrounding landscape as having once been prairie dog colonies. Remnant mounds created by prairie dogs were overgrown with vegetation, but most burrow openings had collapsed or filled with soil.

### 2.2 Site preparation

Prior to capturing prairie dogs at the source colony, the release sites were mowed with a rotary mower powered by a small tractor to ensure that vegetation was of suitable height (i.e., <25 cm). In 2017, 2.61 ha was mowed at the Charlie site, and in 2018, 2.98 ha was mowed at the Charlie's South site. Starter burrows were created using gas‐powered augers with 10‐cm‐diameter drill bits, ranged from 0.9 to 1.2 m in depth, and were angled at approximately 45°. The release sites mimicked the spatial orientation of the source sites and had areas designated for coteries. Each area designated for a coterie was approximately 600 m^2^, with 16 m buffers between coterie extents, and starter burrows within coteries were 8 m apart. In 2017, the Charlie release site accommodated 39 coteries and in 2018, the Charlie's South site accommodated 50 coteries. Within each coterie, there were four starter burrows with single‐opening and four starter burrows with double openings. Previous translocation efforts observed that prairie dogs released into augered burrows with a single opening sometimes covered themselves with soil, whereas prairie dogs released into pre‐existing natural burrows displayed more natural behavior (Dullum et al., 2005), which typically have more than one opening (Cincotta, 1989). Starter burrows with double openings were constructed by using an auger to dig two burrows that intersected. Both single‐opening and double‐opening burrows had chambers at the deepest point of the burrow, which were created by using hand tools to remove soil and dig out a cavity. The chambers in burrows with single openings were approximately 0.3 m^2^ and were large enough to accommodate a minimum of two adult prairie dogs, and the chambers in burrows with double openings were approximately 0.5 m^2^ and were large enough to accommodate a minimum of three adult prairie dogs. Deltamethrin was applied to each burrow and annually thereafter as a plague mitigation strategy (Eads & Biggins, 2019; Seery et al., 2003).

### 2.3 Prairie dog capture

We captured prairie dogs at source colonies that were near Lower Brule, South Dakota, USA. Source colonies were located approximately 48 km away and were undesired because of land use conflicts (i.e., livestock grazing, within highway right‐of‐way). Between August 11 and 19, 2017, weather conditions allowed for five trap days. Between July 7 and 13, 2018, a combination of 4 days of trapping and three partial days of flushing techniques (i.e, burrow flooding) were used to capture individuals. Tomahawk (Hazelhurst, WI, USA) and Tru‐Catch (Belle Fourche, SD, USA) wire cage traps (60 cm × 18 cm × 18 cm and 61 cm × 20 cm × 23 cm) were uniquely marked and deployed in clusters within coteries that allowed for related or neighboring individuals to be captured, held, and released together. Prior to trap placement, we noted patterns in vegetation created by the foraging and clipping behavior of prairie dogs and conducted observations of prairie dogs, which guided the delineation of coterie boundaries and the location of trap clusters. We prebaited traps with a commercially available oat/molasses feed for 3–5 days prior to trapping for all sites to increase capture success (Long et al., 2006).

Flushing provided a viable alternative when leaving traps for extended periods was not an option. The flushing technique is the flooding of active burrows with approximately 200–800 L of water and a small amount of dishwashing detergent (~ 0.25 L per 100 L of water) from a portable water tank and 5.1 cm hose. A small amount of dishwashing detergent is added to create foam that limits the ability of prairie dogs to see and allows them to exit safely. Prairie dogs are captured by hand as they exit the burrow and placed into portable kennels. All prairie dogs captured from one burrow were placed into one kennel. Kennels were marked by coterie to ensure related or neighboring animals were released together.

### 2.4 Processing

Upon capture prairie dogs were kept overnight to be processed the following day. Prairie dogs were kept in kennels in a secure large shelter and provided with grain, lettuce, and carrots. Prairie dogs were anesthetized using isoflurane mixed with oxygen and administered with a vaporizer. Prairie dogs were weighed to a tenth of a gram and implanted with a passive integrated transponder (PIT: AVID® Identification Systems, Norco, CA) tag for individual identification. Sex and age classes (juvenile and adult) were recorded as determined by weight and appearance (Hoogland, 1995). Groups that were trapped and flushed in presumed coterie units based on spatial orientation, were processed sequentially, and placed together into labeled plastic kennels lined with grass hay, carrots, and oats for food. Following processing, animals were taken to the release site later that same day. All methods used were approved by the Department of Wildlife, Fish and Recreation of the Lower Brule Sioux Tribe.

### 2.5 Release

In 2017 and 2018, prairie dogs were released into burrows to maintain the coteries of the source colonies. The spatial orientation of neighboring coteries from the source colonies was also maintained. The locations of all burrows and coteries were recorded with a GPS receiver (Garmin Ltd., Olathe, Kansas). For coteries that had multiple individuals to release, we ensured that individuals were released into both single‐opening burrows and into double‐opening burrows. Up to three individuals were released into single‐opening burrows and up to five individuals were released into double‐opening burrows. Groups typically contained one adult male and the remaining individuals were adult females and juveniles of both sexes. We placed a wire mesh cage (0.8 m × 0.9 m × 0.4 m) over each burrow used for release to provide temporary protection from aerial and terrestrial predators and to discourage immediate dispersal. The acclimation cages were kept over the burrow openings for 3 days, during which grain, lettuce, and carrots were provided. Prairie dogs could easily burrow under the acclimation cages to get out or back in.

### 2.6 Recapture

To sample the population at the release site; animals were recaptured at approximately 1‐month and 1‐year post‐translocation at both release sites to determine retention and to determine if, and how far, animals moved from where they were initially released. The prairie dogs translocated in 2017 (Charlie's colony) were recaptured September 26–28, 2017, at 37–39 days (recapture 1) and August 16–18, 2018, at 361–363 days (recapture 2) post initial release. The prairie dogs translocated in 2018 (Charlie's South colony) were recaptured August 23–25, 2018, at 40–42 days (recapture 1) and August 21–23, 2019, 405–407 days (recapture 2) post initial release. For simplicity of interpretation, we refer to recapture 1 as 1 month and recapture 2 as 1 year throughout the remainder of the manuscript. Traps with individual identification tags were set in a 10 m × 10 m grid across the entire colony where active prairie dog presence was visible. All trap locations within the grid were recorded with a GPS receiver. Traps were prebaited with sweet oat grain mixture for 3 days prior to recapture efforts. Captured animals were weighed, scanned for PIT tag identification number, marked with permanent marker, and released into the nearest burrow. All individuals that did not have PIT tag identifiers were marked with permanent markers, and sex and age recorded. During subsequent trapping days, traps that contained marked individuals were released immediately.

### 2.7 Analyses

#### 2.7.1 Colony expansion

To evaluate colony retention and expansion, we mapped the perimeter of both prairie dog colonies at the time of release and each year until 2021 (Figure 1). The perimeter was the outermost burrows that had clear visual signs of use (e.g., prairie dog present, fresh scat or diggings), which was the augered burrows at the time of release. We input each boundary in ArcGIS v10.7.1 (ESRI, Redlands, CA, USA) and calculated the area of the colony for each year. If the occupied extent of the colony encompassed the same area, or increased, at the end of the study period, we considered this to be an indication of continued success, i.e., that the site had been successfully recolonized or established 3–4 years post‐release.

#### 2.7.2 Distance moved post‐release

To understand whether prairie dogs remained within their coterie as released or whether they moved within the colony and established new territories, we measured the distance from where each prairie dog was initially released to their 1‐month location and from their 1‐month location to their 1‐year location in ArcGIS v10.7.1. Furthermore, we compared the average distance between recapture locations to the diameter of small (0.05 ha), average (0.32 ha), and large (1.01 ha) territory sizes (Hoogland, 2006b) and identified the percent of prairie dogs that were recaptured within the given territory size across each time step. We used chi‐square goodness‐of‐fit tests to determine whether the proportion of prairie dogs that moved in each territory size differed between the Charlie's and Charlie's South colonies. We assumed that the distance between release and 1‐month locations and 1‐month and 1‐year locations could provide a representation of the territory size of their coteries.

#### 2.7.3 Effect of coterie dynamics on retention

To determine whether the number of individuals in a coterie and the age structure and sex composition of the coterie influenced retention, a binary response variable, we used generalized linear mixed models (GLMM; Jamil et al., 2013) in Program R statistical base package (R Development Core Team, Version 3.6.32020). Individuals were released with the coterie they were captured with, therefore, we included coterie as a random effect to account for potential effects of social structure that might differ by coterie and not be explained by our predictor variables. In addition to sex (male or female), age (juvenile or adult), and the group size of the coterie, we also included weight (g), burrow release type (single or double openings), and release site (Charlie's or Charlie's South) as fixed effect predictor variables to account for additional variation. We also created a combined variable of sex and age (adult male or female and juvenile male or female). We did not allow these combined age‐sex variables to enter the same model as age or sex because of the inherent correlation between them. No other variables were correlated at >0.6. Rather than run all possible model combinations, which can result in overly complicated models with reduced biological meaning, we used expert opinion to determine biologically relevant models based on the biology of the system. We also included a global and a null model with just the random effect and intercept. We calculated AIC*c* because of the small sample size, ΔAIC*c*, AIC*c* weight, and log‐likelihood to rank models (Burnham & Anderson, 2002). Models within 2 AIC*c* of the top model were considered indistinguishable from each other (Burnham & Anderson, 2002). Once top models were identified, we calculated model‐averaged parameter estimates with 95% confidence intervals (Arnold, 2010). Parameters whose confidence intervals did not overlap zero were determined to be important predictors or prairie dog recapture success. We repeated this process for 1‐month and 1‐year post‐release.

## 3 RESULTS

### 3.1 Release and recapture

We marked and translocated a total of 658 prairie dogs, 293 in 2017 at Charlie's, and 365 in 2018 in Charlie's South (Table 1). In 2017, between 1 and 23 individuals were released per coterie, with an average of 7.5 (SD ± 4.96) individuals released at each spatial cluster. In 2018, there were 1–20 individuals released per coterie, with an average of 7.5 (SD ± 5.09) individuals released at each spatial cluster. Of the 293 prairie dogs translocated in 2017, 200 (68%) were recaptured 1‐month post‐release, with a sex ratio of adult females to males of 1.05:1, the same as at the time of release. One‐year post‐release, 411 prairie dogs were captured, of which 130 (44%) had been released in 2017 and 281 were unmarked. Of the 281 unmarked individuals, 9 were adults and 272 were juveniles (Table 1). Of the 365 prairie dogs translocated in 2018, 253 (69%) were recaptured 1‐month post‐release with a sex ratio of adult females to males of 1.02:1, nearly the same as at the time of release, which was 1.09:1. At 1‐year post‐release, 365 prairie dogs were captured, of which 128 (36%) were released in 2018 (Table 1). Of the 237 unmarked individuals, 14 were adults and 223 were juveniles. When both colonies were examined as a complex, 69% of the prairie dogs were recaptured 1‐month post‐release and 39% were recaptured 1‐year post‐release (Table 1).

**TABLE 1:** Count and retention rate, as a percent, of marked black‐tailed prairie dogs released with passive integrated transponders (PIT) and recaptured, with and without PIT tags, at approximately 1‐month and 1‐year post‐release by age class and sex for the Charlie's and Charlie's South colonies. Age class and sex are representative of the time at release for translocated individuals and at the time of capture for unmarked individuals

Age class, sex	Counts					% retention of marked		% captured at 1 year compared with released
	Translocated individuals	PIT marked individuals, 1‐month	PIT marked individuals, 1‐year	Unmarked individuals, 1‐month	Unmarked individuals, 1‐year	Release to 1‐month	Release to 1‐year	
2017 Translocation—Charlie's								
Adult, Female	93	60	52	0	4	65%	56%	60%
Adult, Male	88	57	40	0	5	65%	45%	51%
Juvenile, Female	58	39	23	0	130	67%	40%	264%
Juvenile, Male	54	44	15	0	142	81%	28%	291%
Total	293	200	130	0	281	68%	44%	140%
2018 Translocation—Charlie's South								
Adult, Female	83	50	38	1	2	60%	46%	48%
Adult, Male	76	49	33	0	12	64%	43%	59%
Juvenile, Female	94	64	35	1	114	68%	37%	159%
Juvenile, Male	112	90	22	0	109	80%	20%	117%
Total	365	253	128	2	237	69%	35%	100%
Combined sites								
Adult, Female	176	110	90	1	6	63%	51%	55%
Adult, Male	164	106	73	0	17	65%	45%	55%
Juvenile, Female	152	103	58	1	244	68%	38%	199%
Juvenile, Male	166	134	37	0	251	81%	22%	173%
Total	658	453	258	2	518	69%	39%	118%

### 3.2 Colony expansion

Throughout the duration of the study, and beyond, the full extent of the original release site remained occupied, and the overall footprint of the colony expanded (Grassel, personal communication; Figure 1). At the time of release, the Charlie's release site encompassed 2.61 ha. One year later, the colony had expanded to 5.19 ha, grew slightly larger by year two to 5.22 ha, encompassed 5.74 ha in year three, and was 5.75 ha in year four. At Charlie's South, prairie dogs were released across 2.98 ha. One year later, the Charlie's South colony had expanded to 4.2 ha, by year two the site encompassed 5.44 ha, and grew to 6.35 ha in year three. Combined, the two sites expanded from 5.59 ha to 12.10 ha more than twice the size (2.16 times greater) when the final year of mapping occurred.

### 3.3 Distance moved post‐release

**FIGURE 2:**
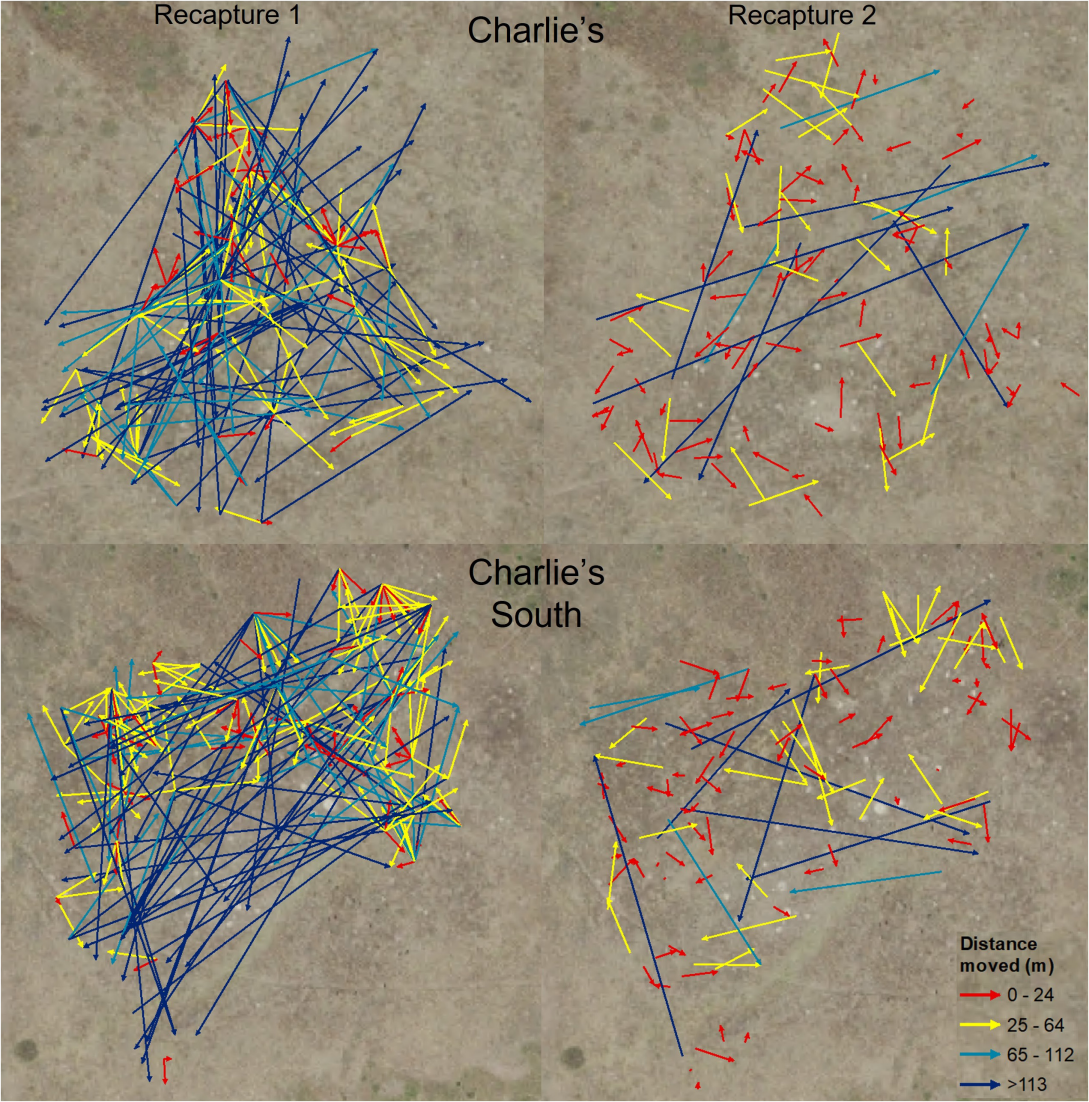
Distance moved by black‐tailed prairie dogs from the release site to approximately 1‐month post‐release, and from 1‐month locations to 1‐year post‐release locations, for Charlie's and Charlie's South sites. Arrows correspond to the direction of movement from release to recapture, and colors correspond with linear distance of small, medium, large, and beyond expected territory sizes maintained by coteries.

There was a marked difference in the distance prairie dogs moved from the time of release to 1 month and from 1 month to 1 year (Table 2, Figure 2). Within territory sizes, prairie dogs did not move significantly different distances between the two colonies at 1 month ($X_{3}^{2}$  = 1.81, *p* = .61) or 1 year ($X_{3}^{2}$  = 1.29, *p* = .73); therefore, we combined the data from both colonies to represent the prairie dog complex. Between the 1‐month recapture and the 1‐year recapture, the percent of individuals that remained within the small‐sized territory (≤24 m) increased from an average of 28% to 67%, decreased within the medium‐sized territory (25–64 m) from 34% to 24%, decreased within the large‐sized territory (65–112 m) from 17% to 3%, and decreased beyond the large territory size (≥ 113 m) from 21% to 6% (Table 2, Figure 2).

**TABLE 2:** Percent of marked black‐tailed paired dogs that were recaptured within defined territory sizes from release to 1‐month post‐release, and from 1‐month to 1‐year for the Charlie's and Charlie's South colonies. Distances are binned based on territory size as defined in Hoogland (2006b)

Distance moved from previous location	Charlie's		Charlie's South	
	1‐month	1‐year	1‐month	1‐year
Small (<24 m)	28%	70%	27%	64%
Medium (25–64 m)	30%	21%	38%	27%
Large (65–112 m)	17%	3%	16%	2%
Beyond (≥113 m)	25%	6%	19%	7%

### 3.4 Distance moved by age sex class

**FIGURE 3:**
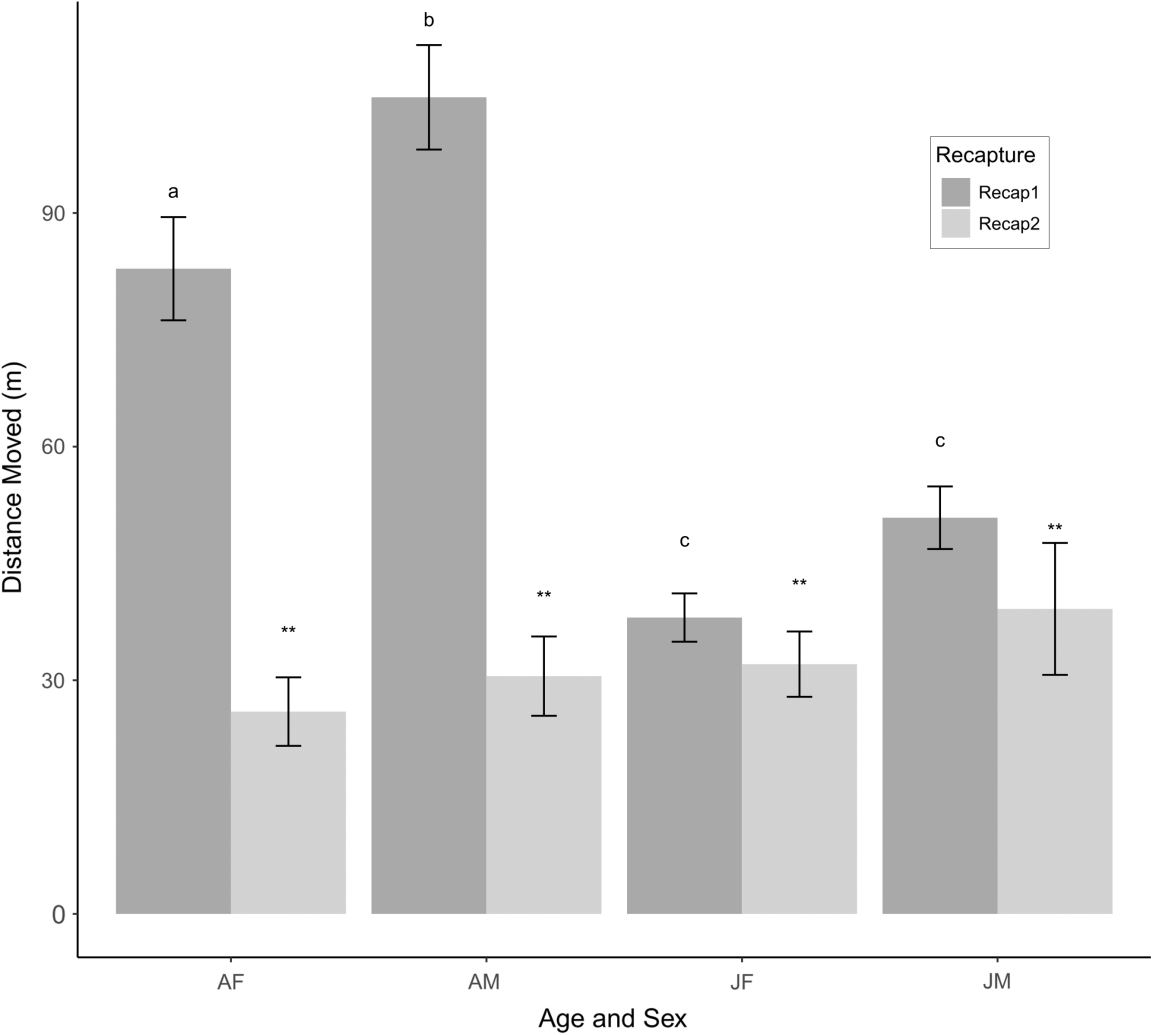
Average distance (m) moved (error bars are standard errors) of prairie dogs by age and sex class for adult females (AF), adult male (AM), juvenile female (JF), and juvenile male (JM) between initial release and 1‐month post‐release locations, and distance between 1‐month locations to 1‐year post‐release locations. Letters and ** indicate significant differences in average distance moved within recapture periods based on the Tukey's pairwise test (*p* < .05).

In a post‐hoc analysis that was deemed valuable after observing the overall distances moved, we also compared the distance moved across age and sex classes using analysis of variance with Tukey's pairwise comparisons to assess whether there were differences between age or sex classes by recapture period. At 1‐month post‐release, there was a significant difference in average distance moved among age and sex classes (*F*_3,448_ = 31.69, *p* < .001). Adult female and adult male prairie dogs were recaptured significantly farther from the coterie they were released at compared with juvenile female or juvenile male prairie dogs (all *p* < .001; adult female: $\overset{―}{x}$ = 82.8 m SE = 6.63 m, adult male: $\overset{―}{x}$ = 104.9 m SE 6.71 m, juvenile female: $\overset{―}{x}$ = 38.1 m SE = 3.10 m, juvenile male: $\overset{―}{x}$ = 50.9 m SE = 4.02 m; Figure 3). In addition, adult males moved further than adult females (*p* = .02), though there was no difference in distance moved between juvenile males and juvenile females (*p =* .30; Figure 3). At 1‐year post‐release, there was not a significant difference in the average distance moved among adult or juvenile prairie dogs (juvenile prairie dogs had become yearlings by this time), regardless of sex (*F*_3,239_ = 0.93, *p* = .43; adult female: $\overset{―}{x}$ = 26.0 m SE = 4.40 m, adult male: $\overset{―}{x}$ = 30.5 m SE = 5.10 m, juvenile female: $\overset{―}{x}$ = 32.1 m SE = 4.19 m, juvenile male: $\overset{―}{x}$ = 39.2 m SE = 8.47 m; Figure 3).

### 3.5 Effect of coterie dynamics on retention

Eight of the 13 models examined were considered top models (<2 AIC*c*) and were similar in describing the retention of PIT‐tagged individuals approximately 1‐month post‐release. Model‐averaged parameter estimates identified sex, age, and sex age class as the only informative parameters with confidence intervals not overlapping zero (Table 3). Males had greater retention than females, and juveniles were more likely to be recaught than adults (Tables 1 and 3). When age and sex were combined as a grouped variable, juvenile males had the greatest retention rates (Table 3). There was no difference between sites, the number of individuals released as a coterie, or whether the animals were released into burrows with one to two openings.

**TABLE 3:** Model averaged parameter estimates for top models (<AIC *c*) estimating retention at 1‐month post‐release and 1‐year post‐release

Parameter	Estimate	SE	CI—Lower	CI—Upper
1‐month post‐release				
Site (CS)	−0.03	0.24	−0.5	0.44
Group size	0.03	0.02	−0.01	0.08
Auger (Single)	0.1	0.18	−0.26	0.45
Sex (Male)^a^	0.4	0.18	0.04	0.75
Age (Juvenile)^a^	0.42	0.19	0.04	0.8
Sex Age Class (AM)	0.16	0.24	−0.31	0.64
Sex Age Class (JF)	0.18	0.26	−0.32	0.68
Sex Age Class (JM)^a^	0.87	0.27	0.34	1.14
1‐year post‐release				
Site (CS)	0.26	0.22	−0.18	0.7
Group Size	0.03	0.02	−0.01	0.06
Auger (Single)	0.04	0.17	−0.3	0.37
Sex (Male)^a^	−0.65	0.17	−0.99	−0.31
Weight^a^	0.18	0.04	0.11	0.25

*Note*: Site, Charlie's or Charlie's South and also controls for year; Sex, male or female; Age, juvenile or adult; Group Size, number of individuals released within the coterie; Sex Age Class, combination of sex and age; Auger, one or two burrow openings; Weight, weight at the time of release.

^a^ Indicates significance where confidence intervals (CI) do not overlap zero.

At 1‐year post‐release, four models were among the top‐ranked models (<2 AIC*c*) in describing retention at the release site. Model‐averaged parameter estimates identified sex and weight as the only informative parameters with confidence intervals not overlapping zero (Table 3). Unlike at 1 month, males displayed lower retention than females, and recapture increased with weight at the time of release (Tables 1 and 3). Similar to the 1‐month post‐release, there was no site‐level effect, the number of individuals released as a coterie, or whether the animals were released into burrows with one or two openings. The combined age and sex variable did not enter the top models during recapture 2 (Table 3).

## 4 DISCUSSION

The efforts to reestablish prairie dog colonies at this site were predicated on the opportunity to reduce human–wildlife conflict where source prairie dogs were present in an undesired location, while simultaneously restoring the species in the desired location. We translocated prairie dogs from source sites and successfully reestablished two prairie dog colonies that had not recovered from losses sustained from the epizootic plague. Mid‐term success was demonstrated by successful recruitment 1‐year post‐release resulting in more prairie dogs being trapped than were released. Continued success was realized through colony occupancy and expansion for at least three and 4 years beyond the time of the translocation. Recognizing that earlier efforts of reestablishing prairie dogs via translocation at the same release site were unsuccessful, we posit that the modifications made in our methodology were beneficial to the success of the translocation. Furthermore, we documented that while the translocation initially disrupted the complex social structure of prairie dog coteries, by 1 year later, it appeared that the colony structure had stabilized.

One year after the translocations, 39% of translocated individuals were recaptured plus an additional 495 unmarked juveniles (272 unmarked juveniles at Charlie's, and 223 unmarked juveniles at Charlie's South) indicating that, at minimum, the population had increased by at least 18%. The numbers of juveniles also illustrate that there was an adequate level of adult survival to result in recruitment and the potential for population persistence into the future. Because prairie dogs have been demonstrated to have larger litters in newer colonies than older ones (Garrett et al., 1982), the site likely offered ample resources that allowed the newly translocated individuals to successfully reproduce. Annual mapping of the site post‐release confirmed that the entirety of the release site remained occupied, and that the colony extent had more than doubled three‐ and four‐year post‐release. We used colony extent and visual confirmation that the entirety of the release site had active prairie dog presence because colony area is the metric commonly used in management; however, we recognize that colony expansion does not quantify prairie dog densities or population size (Bruggeman & Licht, 2020).

While there was only one unmarked adult that was captured at 1‐month post‐release, there were nearly two dozen unmarked adults that were captured at 1‐year recapture event. The unmarked adults captured may indicate immigration into the colony, or that individuals lost pit tags, which is rare but possible. If these unmarked individuals were immigrants, this could be an early indication that the reestablishment of these two colonies may contribute to the restoration and connectivity of the Fort Hale Bottom prairie dog complex. While beyond the scope of this study, further research evaluating how newly translocated colonies may influence the dispersal dynamics of conspecifics from surrounding colonies would add understanding to the composition and functioning of prairie dog complexes.

We followed translocation recommendations to utilize areas that were previously occupied by prairie dogs and had visible evidence of past occupation (Long et al., 2006; Truett et al., 2001). Additionally, the site was located within proximity to other colonies meeting dispersal and complex criteria; and a plan was in place to mitigate the initial reason the site was extirpated, by managing the site for sylvatic plague following the translocation efforts. However, because the previous translocation effort was considered unsuccessful and our selected release site had natural burrows with collapsed or filled‐in openings; we made three modifications to our methodology: adding chambers in augered burrows, utilization of acclimation cages, and maintaining coteries at release burrows. Release site preparation often requires the installation of artificial burrows and nest boxes with the use of heavy equipment (Bly‐Honness et al., 2004; Curtis et al., 2014; Davidson et al., 2014) or the usage of abandoned pre‐existing burrows (Long et al., 2006, Truett et al., 2001). Neither of these options were available to us due to the preclusion of using heavy equipment necessary to install artificial burrows and nest boxes and there were no pre‐existing burrows that were in good condition.

Soils have been identified as one of the most important variables for the translocation of other colonial, burrowing mammals (Swaisgood et al., 2019), and the soils at our site were highly conducive for effectively making temporary release burrows with the use of augers, which is not applicable to all translocation sites. The silty loam soil type at the release site allowed for using augers to create starter burrows and for using hand tools to chisel out chambers and remove excess soil at the deepest part of each augered hole. While the past failed translocation utilized augered starter burrows, this current effort included the creation of chambers that may have been more hospitable to animals upon initial release. Past translocation efforts at other sites have observed prairie dogs rarely using augered starter burrows for longer than a few days (Truett et al., 2001, Sterling Krank, personal observation). Likewise, we observed that when some prairie dogs entered augered burrows, they dug beyond the predefined burrow confines and instead reemerged from newly excavated burrows. We also observed that released prairie dogs were able to open old natural burrows within days of being released and may indicate that those old networks underground were valuable.

Other species of translocated colonial ground squirrels have been observed to settle outside of release sites (Van Vuren et al., 1997), and acclimation cages have been utilized to enhance the success of translocations of other small mammal species as well (Shier et al., 2021; Wang & Shier, 2021). While not used in the previous unsuccessful effort at our site, acclimation cages were used during this translocation effort as has been recommended for prairie dogs (Long et al., 2006; Roe & Roe, 2004). Footage obtained from remotely triggered game cameras that were opportunistically placed at some of the release burrows showed several prairie dogs burrowing out of the acclimation cages within hours of release but also showed these prairie dogs returning to the acclimation cages repeatedly even when they had the ability to leave the release burrows. Anecdotally, the temporary provision of acclimation cages may have provided translocated prairie dogs refuge and the footage captured seemed to show that individuals did not bolt from the release site when able to. Additionally, the supplemental food that was provided while acclimation cages were in use was observed to be consumed by the prairie dogs. Recognizing that rapid dispersal of animals from release sites is common, our utilization of acclimation cages with supplemental food was incorporated as a best practice for prairie dogs. Based on the video footage and visual evidence of translocated individuals utilizing the acclimation cages, this suggests that using soft‐release techniques is likely beneficial for prairie dog translocations.

Another modification to our methodology was an increased consideration of social structure, as we released individuals with presumed coteries unlike the previous failed attempt. Past research has shown mixed results for the added benefit releasing prairie dogs as family units have on survival. For example, Shier (2006) found that prairie dogs translocated with family units increased survival by a factor of five and females had higher reproductive success, but Bly‐Honness et al. (2004) suggested the maintenance of coterie unity during translocation did not enhance post‐release survival. While we did not examine survival in this study, we did examine what factors influenced site retention. We did not observe an influence of coterie size on retention, though we acknowledge this was not a paired design study with a control group that was not released as a coterie to compare to as that was not a primary goal of this translocation. We did, however, observe that while juvenile males had the highest retention rates 1‐month post‐release they had the greatest decline in retention by 1‐year post‐release, whereas adult retention remained relatively stable throughout. Furthermore, adult females and heavier individuals at the time of release also had higher retention rates. Juvenile prairie dogs are less likely than adults to survive until the following spring (Hoogland, 2006c), which may account for the observed reduction in retention. Additionally, adult females tend to have high site philopatry and heavier prairie dogs, regardless of sex, are more likely to survive overwinter (Hoogland, 2006c), which aligns with the observations in this study as well.

To our knowledge, this is the first study that has measured the distance moved by prairie dogs after a translocation, further enhancing our understanding of prairie dog social dynamics post‐translocation. Interestingly, and not as hypothesized, our spatial analysis provided mixed support that prairie dogs remained at the release area designated for their respective coteries. While only 28% of the translocated prairie dogs were recaptured within the small territory size 1‐month post‐release, 67% of the prairie dogs that were recaptured 1 year later were still within the small territory size of where they were at 1‐month post‐release. Conversely, 21% of the prairie dogs recaptured at 1 month moved further than the large territory size and just 6% of the recaptured prairie dogs at 1 year had made such considerable movements. This indicates that prairie dogs had largely established new territories 1‐month post‐release from where they were initially released, and still maintained those new territories 1 year later. Even with greater initial movement than expected, these movements were still within the footprint of the release site providing further support for our overarching objective of establishing prairie dog colonies through translocation.

Translocation is an initially disruptive event, and in this case, the greatest movements were by adults during the first‐month post‐release. Both male and female adult prairie dogs moved large distances immediately after release, more than twice the average distance moved by juveniles. The different distances moved by age and sex and the directional movements observed radiating from a coterie rather than in a uniform direction (Figure 2), suggests that entire coteries did not shift as a cohesive unit. We can only further hypothesize as to why this is, but one possibility is that adults are more territorial than juveniles and are more likely to venture further to find familiar territory. California ground squirrels have been observed to make large movements in search of familiar territory post‐translocation, with 46% of surviving individuals returning to their original territory over the course of 1–16 days (Van Vuren et al., 1997). It was not possible for the prairie dogs observed during our study to return to their original territory because of the extensive distance between capture and release sites, but it is possible that individuals may have exhibited homing movements when acclimation cages were released. Nevertheless, this disruption appears to be short‐lived—in that 91% of all prairie dogs that were recaptured 1‐year post‐release were within the small‐ and medium‐sized territories of where they were 1‐month post‐release—and importantly, the release site remained occupied and expanded.

Territory sizes maintained by coteries vary and are likely dependent on a suite of variables that can be traced to ecological site conditions such as soil type, temperature, precipitation and ultimately plant productivity (Geaumont et al., 2016) and demographic features (Kusch & Lane, 2021). We used previously identified small, medium, and large territory size ranges (Hoogland, 2006b) as the basis for assessing whether prairie dogs remained within a given territory size. We did not have previous knowledge of territory size for prairie dogs in this habitat under the site conditions at the time and it is possible that the sizes we evaluated were not representative. Given the wide range of territory sizes, we evaluated the difference in initial movements and secondary movements; we are confident that we adequately captured the range of possibilities. Recent research, however, suggests that the social structure of prairie dogs may be more variable than previously thought; with dispersal across sex and age classes, and high coterie structure variability in a nontranslocated colony (Kusch et al., 2020; Kusch & Lane, 2021). Additionally, our designation of presumed coterie delineations at the source site was based on behavioral observations and vegetation clipping patterns, and while we have confidence in our classification, it is also important to recognize that post‐translocation movements may also be reflective of prairie dogs reorganizing with their original social units that were misidentified. We are not suggesting there is not an added benefit of relocating prairie dogs with the coteries they are captured with, that was beyond the scope of this study, only that prairie dogs appeared to break their coterie structure that we observed and establish new territories upon release. The importance of social structure and spatial release dynamics of neighbor conspecifics has been demonstrated to impact the survival and reproductive fitness even of a solitary species when translocated (Shier & Swaisgood, 2011) and is particularly salient for highly social species as well (Goldenberg et al., 2019; Shier, 2006). Because our translocation effort met our definition of success, we recommend keeping coteries intact during capture and release, as this was one key modification to our methodology following the earlier failed translocation.

## 5 CONCLUSION

Sylvatic plague can result in widespread and dramatic population losses of prairie dogs; therefore, wildlife managers and conservation practitioners currently rely on plague mitigation tools and translocations to effectively maintain the prairie dog ecosystem. Translocations of other mammals with the intention of restoring ecological processes, including many identified with the role that keystone species play, are increasingly being identified as a specific goal for translocation programs (Palmer et al., 2020; Swaisgood et al., 2019; Watson & Watson, 2015). An intended outcome of reestablishing prairie dogs at this site was to facilitate the ecological role that the species plays in North American grasslands. Nesting burrowing owls, ferruginous hawks, badgers, lekking sharp‐tailed grouse (*Tympanuchus phasianellus*), and species of shortgrass prairie songbirds including horned lark (*Eremophila alpestris*) and killdeer (*Charadrius vociferus*) were all observed utilizing the new colonies (Guernsey and Grassel, personal observation). Anecdotally, this indicates that the reestablishment of prairie dogs can provide important habitat and prey base for many grassland species. We anticipate with further growth and recovery of these colonies—as part of a larger prairie dog complex—the site will likely support the critically endangered black‐footed ferret as it once did.

Prairie dog conservation presents challenges because of the important ecological role that they play while simultaneously being undesired in many working landscapes. The Lower Brule Sioux Tribe Department of Fish, Wildlife, and Recreation has multiple objectives that include the restoration and maintenance of native wildlife species, such as the endangered black‐footed ferret, an obligate prairie dog‐dependent species. Additional objectives include addressing the concerns of constituents, which includes those that desire the removal of, or decreased densities, of prairie dogs. Trapping efforts on source colonies included areas along roadways, small pastures grazed by livestock, and near a private residence and resulted in a reduced number of prairie dogs that would ultimately be controlled. While translocation likely does not entirely mitigate conflicts with all constituents, the tool can be used to show that concerns are heard, and that management and conservation objectives can both benefit from coordinated efforts to reduce conflict. For sites like Lower Brule Sioux Tribal lands that have dual management priorities that include working towards the recovery of black‐footed ferrets and employing conflict mitigation for prairie dogs, this research supports that translocation can be a tool to dually address both needs.

## AUTHOR CONTRIBUTIONS

**Noelle C. Guernsey:** Conceptualization (lead); formal analysis (supporting); investigation (equal); methodology (equal); project administration (lead); resources (supporting); visualization (supporting); writing – original draft (lead); writing – review and editing (equal). **Patrick E. Lendrum:** Conceptualization (supporting); formal analysis (lead); investigation (equal); methodology (equal); visualization (lead); writing – original draft (supporting); writing – review and editing (equal). **Lindsey Sterling Krank:** Conceptualization (supporting); funding acquisition (lead); investigation (equal); methodology (equal); project administration (supporting); resources (supporting); writing – review and editing (equal). **Shaun M. Grassel:** Conceptualization (supporting); formal analysis (supporting); funding acquisition (supporting); investigation (equal); methodology (equal); project administration (supporting); resources (lead); writing – review and editing (equal).

## CONFLICT OF INTEREST

There are no competing interests.

## Data Availability

Upon acceptance, data will be made available in a publicly accessible repository such as Dryad.
